# Prognostic value of plasma secretoneurin concentration in patients with heart failure with reduced ejection fraction in one-year follow-up

**DOI:** 10.1080/07853890.2024.2305309

**Published:** 2024-01-23

**Authors:** Łukasz Wołowiec, Daniel Rogowicz, Jacek Budzyński, Joanna Banach, Anna Wołowiec, Mariusz Kozakiewicz, Maciej Bieliński, Albert Jaśniak, Joanna Osiak, Grzegorz Grześk

**Affiliations:** a Department of Cardiology and Clinical Pharmacology, Faculty of Health Sciences, Ludwik Rydygier Collegium Medicum in Bydgoszcz, Nicolaus Copernicus University, Toruń, Poland; b Department of Vascular and Internal Diseases, Collegium Medicum in Bydgoszcz, Nicolaus Copernicus University, Toruń, Poland; c Department of Geriatrics, Division of Biochemistry and Biogerontology, Collegium Medicum in Bydgoszcz, Nicolaus Copernicus University, Toruń, Poland; d Department of Clinical Neuropsychology, Nicolaus Copernicus University in Toruń, Collegium Medicum in Bydgoszcz, Bydgoszcz, Poland

**Keywords:** Secretoneurin, heart failure, HFrEF, biomarker, cardiovascular system

## Abstract

**Background:**

This is the first study to examine the clinical utility of measuring plasma secretoneurin (SN) levels in patients with heart failure with reduced ejection fraction (HFrEF), as a predictor of unplanned hospitalization, and all-cause mortality independently, and as a composite endpoint at one-year follow-up.

**Methods:**

The study group includes 124 caucasian patients in New York Heart Association (NYHA) classes II to IV. Plasma SN concentrations were statistically analyzed in relation to sex, age, BMI, etiology of HFrEF, pharmacotherapy, clinical, laboratory and echocardiographic parameters. Samples were collected within 24 h of admission to the hospital.

**Key results:**

In the 12-month follow-up, high SN levels were noted for all three endpoints.

**Conclusions:**

SN positively correlates with HF severity measured by NYHA classes and proves to be a useful prognostic parameter in predicting unplanned hospitalizations and all-cause mortality among patients with HFrEF. Patients with high SN levels may benefit from systematic follow-up and may be candidates for more aggressive treatment.

## 1. Introduction

SN belongs to the secretogranin/chromogranin family of peptides found in cardiomyocytes and neuroendocrine tissue. It is a highly conserved product of the proteolytic cleavage of the precursor protein, secretogranin II (SgII, also known as chromogranin C). SN increases in cardiac cells during myocardial dysfunction, exerting cardioprotective effects by protecting cardiomyocytes from ischemia-reperfusion injury, apoptosis, arrhythmogenesis, and myocardial remodeling [1]. SN as a potential cardiovascular marker increases its concentration in various clinical situations, providing prognostic information in the course of heart failure (HF), acute coronary syndrome, ventricular arrhythmias, cardiac surgeries, cardiac arrest, as well as in severe sepsis and septic shock [2–8]. The extensive development of mechanistic research on SN indicates that it operates in separate pathways than commonly used CV markers such as N-terminal fragment of B-type natriuretic propeptide (NT-proBNP) and troponins. This creates an opportunity to search for a potential clinical advantage of using SN over existing markers. Its key effects are thought to be mediated by calcium/calmodulin-dependent kinase II (CaMKII) [5]. CaMKII is a regulator of cellular calcium transport - a key ion in excitation-contraction coupling and intracellular signaling. Dysregulation of cardiomyocyte calcium metabolism is a common element in a wide range of cardiac pathologies, including HFrEF [5]. Inhibition of CaMKII by SN stops uncontrolled calcium leakage from the sarcoplasmic reticulum *via* the ryanodine receptor, which reduces the risk of arrhythmias and calcium-dependent remodeling in HF [1]. SN is probably upregulated as a mechanism compensating for the disturbed calcium metabolism of the cardiomyocyte due to excessive activation of CaMKII [1, 6]. Because of the above reports and the significant role of previously used biomarkers in cardiovascular risk stratification, we decided to evaluate the prognostic value of measuring plasma SN concentration among patients with HFrEF as a predictor of unplanned hospitalization and death from all causes independently and as a composite endpoint in one-year follow-up.

## 2. Aim of the study

The main aim of our study was to assess the usefulness of determining the plasma SN concentration collected within 24 h of admission to the hospital as a predictor of the unplanned hospitalization and all-cause mortality independently and as a CE in the group of patients with HFrEF in a one-year follow-up.

## 3. Methods

### 3.1. Study population

The study group consisted of 124 caucasian patients with HFrEF in NYHA class II-IV, who were either treated on an outpatient basis or hospitalized in the Department of Cardiology and Clinical Pharmacology or Department of Vascular and Internal Diseases at the Nicolaus Copernicus University Collegium Medicum University Hospital No. 2 in Bydgoszcz for planned medical procedures, some of which were on the elective list of patients waiting for heart transplantation. Outpatient patients were under the care of the Cardiology Clinic and Heart Failure Clinic operating at the department. The diagnosis of HFrEF was based on the criteria of the European Society of Cardiology (ESC). Patients received optimal pharmacological treatment for each patient in accordance with the ESC guidelines. The study protocol was approved by the Bioethical Committee of the Nicolaus Copernicus University in Toruń at the Collegium Medicum in Bydgoszcz. Each patient signed an informed consent form after obtaining detailed information about the purpose and scope of the study. The criteria for inclusion in the study were age over 18, heart failure diagnosed according to the criteria included in the guidelines European Society of Cardiology, heart failure class II-IV according to NYHA, left ventricular ejection fraction (LVEF) assessed during the current hospitalization or up to the 6th months earlier ≤ 40%. The exclusion criteria were sepsis or shock from any cause on admission to hospital, acute coronary syndrome, recent (<3 months) myocardial infarction or stroke, active neoplasm, autoimmune diseases, impaired liver function (INR without oral anticoagulation >1.5 or bilirubin total >1.5 mg% or 3 times the upper limit of normal for ALT), corticosteroid therapy, decompensated diabetes mellitus requiring treatment with intravenous insulin infusion, chronic inflammatory bowel diseases, recent (<3 months) surgery.

### 3.2. Secretoneurin determination

All biochemical analytes were routinely collected upon admission to the Department of Cardiology and Clinical Pharmacology. Blood specimens were collected by venipuncture into 5 mL tubes containing tripotassium EDTA. Plasma blood was centrifuged (3000 g for 15 min) and aliquoted into Eppendorf Tubes. Samples were stored at a temperature of −80 °C until biochemical analysis was performed. The plasma SN concentration was measured by an enzyme immunoassay (ELISA) commercial kit SN, ELISA Kit, extraction-free for human, Penisula Laboratories International Inc, catalog number RS-1387.0001, average IC50: 4 ng/ml, range: 0-50ng/ml. According to the manufacturer’s comments, sample extraction was not required for human plasma. Sample were not dilated, reproducibility intra-assay: CV < 10%, and inter-assay: CV < 15%.The table presents the results obtained from patients, expressed in ng/ml and pmol/L. According to the manufacturer, the reactivity with human SN is 100%. The analytical sensitivity of the method (lower detection limit for the test) is 0.5 ng/ml. The results were obtained with SPECTROstarNano, BMG LABTECH spectrophotometric reader using MARS data analysis software version 2.41. The marker was evaluated at 450 nm wavelength. The results were read from the calibration curve prepared for the analyzer used in the study. All analyses were performed in accordance with the manufacturer’s instructions.

### 3.3. NT-proBNP measurement

The samples used were heparinized plasma and were measured using an electrochemiluminescence immunoassay (Elecsys, Roche Diagnostics, Meylan, France).

### 3.4. Statistical analysis

Statistical analysis was conducted using the licensed version of the statistical analysis software STATISTICA version 13.1 (TIBCO Software, Inc., 2017). The statistical significance level was set at a p-value of < 0.05. The normal distribution of the study variables was analyzed using the Kolmogorov-Smirnov test. The results were presented as the mean ± standard deviation; median, interquartile range (IQR); or as a frequency (n, %) of the categorical variables. The statistical significance of differences between groups was verified using the Student’s t-test, the Mann-Whitney U-test, and one-factorial ANOVA with Bonferroni post-hoc test for quantitative variables (when more than one comparison was necessary) and the Chi-square test for qualitative variables. We also used a ROC curve with the lowest Youden’s index and the AUC to determine the cut-off values of the parameters measured. Kaplan Meir analysis was carried out to determine the factors affecting the risk of all-cause mortality and all- cause readmission. Spearman’s correlation was also used. Logistic regression method was applied to determine the risk of measured outcome occurrence associated with respective cut-offs of parameters studied.

## 4. Results

### 4.1. Clinical characteristics

Out of 124 consecutive HFrEF patients, 72 of them were admitted to the hospital with exacerbation (including de novo heart failure), and 52 in stable condition. 122 patients were included in the final statistical analysis because follow-up failed in 2 participants (vure 1).

**Figure 1.**
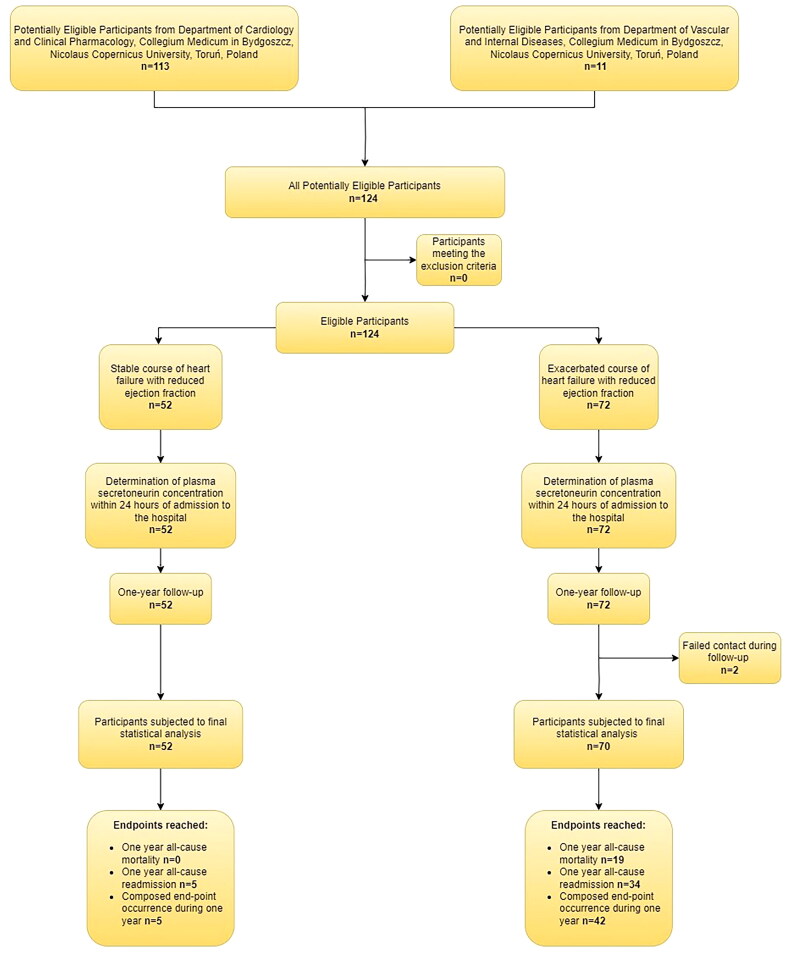
STARD diagram to report flow of participants through the study.

Planned hospitalizations for patients with HF result from the fact that our clinic is a regional center for HF treatment. The planned visits concerned cardiological diagnostics, including cardiac angiography, cardiac catheterization, intracardiac echocardiography or qualification for cardiac implantable electronic devices (CEID) implantation, ablation, or heart transplant.

The etiology of HFrEF in the study group included in the final statistical analysis (*n* = 122) was due to ischemic cardiomyopathy (ICM, *n* = 50) or dilated cardiomyopathy (DCM, *n* = 72). Distribution of etiology in the group with stable HF course: ICM *n* = 16, DCM *n* = 36, while in the group with HF exacerbation: ICM *n* = 36, DMC *n* = 34. Although there is no precise definition of heart failure exacerbation, we decided to include these patients who demonstrated certain dynamics of increasing hypervolaemia, ultimately urging patient to search for medical care. Low-output states were excluded from the study as in our opinion in initial stages it is difficult to make a clear distinction between plain HF exacerbation with hypotension and developing cardiogenic shock being a classical form of acute heart failure. Patients with chronic heart failure (CHF) exacerbation at the day of enrollment were significantly older, had lower TAPSE, blood NT-proBNP and hemoglobin concentrations, as well as higher concentrations of SN, troponin T, creatinine, glucose, and higher RDW, and neutrocytes count (Table 1).

**Table 1.** Baseline data obtained in patients enclosed with and without HFrEF exacerbation.

Parameter	Exacerbation (*n* = 70)	Stable (*n* = 52)	p
Male gender (n, %)	48 (50.50)	47 (49. 47)	0.005
Age (years)	67.93 ± 14.38	51.58 ± 9.16	< 0.001
30-days all-cause mortality (n, %)	5 (7.14)	0	1.0
30-days all-cause readmission (n, %)	59 (15.7)	13 (25.00)	1.0
One year all-cause mortality (n, %)	19 (27.14)	0	<0.001
One year all-cause readmission (n, %)	34 (48.57)	5 (9.62)	<0.001
Composed end-point occurrence during one year (n, %)	42 (84.29)	5 (9.62)	< 0.001
TAPSE (mm)	16.99 ± 3.63	20.83 ± 3.93	<0.001
LVEF (%)	25.66 ± 9.07	28.65 ± 7.53	0.055
Etiology, DCM/ICM (n, %)	34 (48.57)	36 (69.26)	0.022
	36 (51.46)	16 (30.77)	
BMI (kg/m^2^)	29.78 ± 6.55	28.73 ± 4.99	0.335
Diabetes mellitus (n, %)	35 (50.00)	15 (28.85)	0.019
Hypertension (n, %)	47 (67.14)	18 (34.62)	<0.001
Atrial fibrillation (n, %)	40 (57.14)	20 (38.46)	0.071
*c*ardiac implantable electronic devices (n, %)	36 (51.43)	27 (48.08)	<0.001
NT-proBNP (pg/ml)	6373.0 (3345.0 − 12364.0)	441.5 (179.5-1093.5)	<0.001
Secretoneurin (pmol/l)	71.20 (58.15-101.47)	9.11 (6.17-12.74)	<0.001
Troponin T (ng/ml)	0.04 (0.03-0.06)	0.02 (0.01-0.02)	<0.001
Creatinine (mg/dl)	1.34 ± 0.44	1.01 ± 0.27	<0.001
Glucose (mg/dl)	141.69 ± 50.29	110.89 ± 25.72	<0.001
hsCRP (mg/dl)	20.51 ± 34.60	3.98 ± 14.01	0.002
Hemoglobin (g/dl)	12.53 ± 2.23	14.64 ± 1.22	<0.001
White blood cells count (G/L)	8.68 ± 2.81	7.31 ± 1.89	0.003

Data presented as median ± standard deviation, or median (IQR), depending on variable.

distribution. TAPSE: tricuspid annular plane systolic excursion; LVEF: left ventricular ejection fraction; DCM/ICM: dilated cardiomyopathy/ischemic cardiomyopathy; BMI: body mass index; NT-proBNP: N-terminal fragment of B-type natriuretic propeptide; hsCRP: high-sensitivity C-reactive protein; RDW: red cell distribution; p: probability.

Pharmacotherapy differed between groups, mainly in relation to diuretics use. Pharmacotherapy does not include some of the currently used new generation drugs, such as sodium-glucose transport protein 2 (SGLT2) inhibitor, or angiotensin receptor-neprilysin inhibitor (ARNi), because these drugs were not widely available in clinical practice during the years of patient recruitment for the study. Some patients also refused to undergo modern treatment for other reasons, including financial ones. Our patients differed also in relation to CHF severity, expressed as NYHA class (Table 2).

**Table 2.** Values of parameters studied in patients with respective NYHA class.

Parameter	NYHA II (*n* = 39)	NYHA III (*n* = 44)	NYHA IV (*n* = 39)	P23	P24	P34
Male gender (n, %)	37 (94.87)	30 (68.18)	28 (71.79)	0.002	0.006	0.724
Age (years)	50.59 ± 9.68	64.795 ± 15.05	67.00 ± 13.55	< 0.001	< 0.001	0.487
Death during 12 months (n, %)	0	6 (13.64)	13 (33.33)	0.016	< 0.001	0.033
One year readmission (n, %)	5 (12.82)	12 (27.27)	22 (56.41)	0.106	< 0.001	0.007
Composed end-point occurrence during one year (n, %)	5 (12.82)	15 (34.09)	27 (69.27)	0.02	< 0.001	< 0.001
TAPSE (mm)	20.41 ± 3.92	19.02 ± 4.30	16.385 ± 3.37	0.130	< 0.001	0.003
LVEF (%)	29.46 ± 6.83	27.07 ± 8.83	24.26 ± 9.15	0.175	0.006	0.159
Etiology, DCM/ICM (n, %)	25 (64.10)	27 (61.36)	18 (46.15)	0.800	0.114	0.169
	14 (35.90)	17 (38.64)	21 (53.85)			
BMI (kg/m^2^)	28.72 ± 5.48	30.47 ± 6.40	28.67 ± 5.81	0.187	0.973	0.187
Diabetes mellitus (n, %)	9 (23.08)	24 (54.55)	17 (43.59)	0.003	0.056	0.325
Hypertension (n, %)	14 (35.90)	25 (56.82)	26 (66.67)	0.058	0.006	0.364
Atrial fibrillation (n, %)	17 (43.59)	20 (45.45)	23 (58.97)	0.356	0.179	0.325
Cardiac implantable electronic devices (n, %)	19 (48.72)	26 (59.19)	23 (58.97)	0.310	0.330	0.938
NT-proBNP (pg/ml)	373.0 (168.0-1067.0)	5179.0 (1496.5-9429.5)	6173.0 (2986.0-13052.0)	< 0.001	< 0.001	0.366
Secretoneurin (pmol/l)	9.6 (6.5-12.82)	64.8 (14.5-84.2)	68.35 (57.5-103.3)	< 0.001	< 0.001	0.024
Troponin T (ng/ml)	0.01 (0.01-0.02)	0.03 (0.02-0.06)	0.03 (0.03-0.06)	0.009	0.002	0.651
Creatinine (mg/dl)	1.02 ± 0.23	1.21 ± 0.40	1.38 ± 0.48	0.009	< 0.001	0.090
Glucose (mg/dl)	110.54 ± 26.47	132.48 ± 43.04	142.15 ± 53.73	0.007	0.001	0.366
hsCRP (mg/dl)	4.46 ± 16.16	9.59 ± 14.22	26.84 ± 43.34	0.128	0.003	0.015
Hemoglobin (g/dl)	14.65 ± 1.25	13.10 ± 2.20	12.59 ± 2.25	< 0.001	< 0.001	0.302
White blood cells count (1000/mm3)	7.25 ± 1.79	7.77 ± 2.12	9.31 ± 3.16	0.238	0.001	0.010

Data presented as median ± standard deviation, or median (IQR), depending on variable distribution. TAPSE: tricuspid annular plane systolic excursion; LVEF: left ventricular ejection fraction; DCM/ICM: dilated cardiomyopathy/ischemic cardiomyopathy; BMI: body mass index; NT-proBNP: N-terminal fragment of B-type natriuretic propeptide; hsCRP: high-sensitivity C-reactive protein; RDW: red cell distribution.

### 4.2. Comparison of HFrEF patients in relation to measured outcome occurrence

HFrEF patients who died during 12 months of follow-up were significantly older, had lower BMI, lower hematocrit, platelets count, and neutrophils percentage, as well as higher blood concentration of: SN, NT-proBNP, creatinine, and lower blood concentration of hemoglobin than HFrEF patients who survived follow-up period (Table 3).

**Table 3.** Clinical characteristics of patients studied who died during a 12 month follow-up in comparison to those who survived this period.

Parameter	Died (*n* = 19)	Survived (*n* = 103)	p
Male gender (n, %)	14 (73.68)	81 (78.64)	0.636
Age (years)	72.32 ± 11.98	58.86 ± 14.36	< 0.001
NYHA class (II, III vs IV)	0	39 (37.86)	0.115
	6 (31.58)	38 (36.89)	
	13 (68.42)	26 (25.24)	
All-cause read­mission (n, %)	11 (57.89)	28 (27.18)	< 0.01
TAPSE (mm)	16.89 ± 2.92	18.94 ± 4.33	0.051
LVEF (%)	25.26 ± 8.07	27.24 ± 8.63	0.356
Etiology, DCM/ICM (n, %)	9 (47.37)	61 (59.22)	0.341
	10 (52.63)	42 (40.78)	
BMI (kg/m^2^)	25.85 ± 8.07	29.98 ± 5.82	< 0.01
Diabetes mellitus (n, %)	8 (42.11)	42 (40.78)	0.915
Hypertension (n, %)	10 (52.63)	55 (53.40)	0.951
Atrial fibrillation (n, %)	12 (63.16)	48 (46.60)	0.620
Cardiac implantable electronic devices (n, %)	11 (57.89)	77 (74.76)	< 0.01
NT-proBNP (pg/ml)	6712.0 (3244.0-12767.5)	1143.0 (348.0-4756.0)	< 0.001
Secretoneurin (pmol/l)	68.28 (46.2-101.6)	13.23 (8.25-61.01)	< 0.001
Troponin T (ng/ml)	0.04 (0.03-0.06)	0.02 (0.01-0.03)	0.074
Creatinine (mg/dl)	1.43 ± 0.39	1.16 ± 0.40	< 0.01
Glucose (mg/dl)	146.26 ± 56.01	125.29 ± 41.18	0.057
hsCRP (mg/dl)	29.54 ± 37.73	10.50 ± 26.07	< 0.01
Hemoglobin (g/dl)	11.75 ± 2.43	13.74 ± 1.93	< 0.001
White blood cells count (1000/mm3)	8.87 ± 2.91	7.95 ± 2.46	0.151

TAPSE: tricuspid annular plane systolic excursion; LVEF: left ventricular ejection fraction; DCM/ICM: dilated cardiomyopathy/ischemic cardiomyopathy; BMI: body mass index; NT-proBNP: N-terminal fragment of B-type natriuretic propeptide; hsCRP: high-sensitivity C-reactive protein; RDW: red cell distribution; p: probability.

We did not reveal statistically significant differences between groups in regard to known prognostic factors in CHF patients, such as: LVED, TAPSE, troponin T, hsCRP, natrium and kalium. Both groups of patients were treated similarly, both in relation pharmacotherapy and use of CIEDs.

Plasma SN concentration increased with NYHA class, both in ANOVA analysis, and in Spearmen’s correlation (*R* = 0.67; *p* < 0.001). It seems to be worth to underline that correlation coefficient for NYHA class was even higher for SN than for NT-proBNP (*R* = 0.66; *p* < 0.001). Blood SN concentration correlated significantly with blood NT-proBNP concentration (*R* = 0.67; *p* < 0.001). A comparison of the Spearman correlations obtained for SN and NT-proBNP is presented in Table 4.

**Table 4.** A comparison of the Spearman correlations obtained for SN and NT-proBNP.

Clinical feature	NT-proBNP (pmol/l)		Secretoneurin (pmol/l)	
	R Spearman	p	R Spearman	p
Male gender	−0.259	0.004	−0.228	0.011
Age (years)	0.590	< 0.001	0.586	< 0.001
Exacerbated vs. stable course of CHF during follow-up	−0.772	< 0.001	−0.857	< 0.001
NYHA class	0.659	< 0.001	0.672	< 0.001
Need of hospitalization due to CHF exacerbation	0.349	< 0.001	0.288	0.001
Death during follow-up	0.470	< 0.001	0.426	< 0.001
Composite end-point occurrence	0.534	< 0.001	0.465	< 0.001
TAPSE (mm)	−0.487	< 0.001	−0.454	< 0.001
LVEF (%)	−0.272	0.002	−0.107	0.240
BMI (kg/m^2^)	−0.143	0.116	0.089	0.331
History of DM	0.098	0.281	0.215	0.018
History of HT	0.101	0.267	0.291	0.001
History of AF	0.142	0.119	0.204	0.024
Creatinine (mg/dl)	0.419	0.000	0.425	0.000
hs-CRP (mg/dl)	0.569	0.000	0.581	0.000

NT-proBNP: N-terminal fragment of B-type natriuretic propeptide; HFrEF: heart failure with reduced ejection fraction; NYHA: New York Heart Association; TAPSE: tricuspid annular plane systolic excursion; LVEF: left ventricular ejection fraction; BMI: body mass index; DM: diabetes mellitus; HT: hypertension; AF: atrial fibrillation; hs-CRP: high-sensitivity C-reactive protein.

When HFrEF patients studied were divided in relation to the need of rehospitalization due to any cause, readmitted patients had, i.a., significantly higher blood SN concentration than those who remained stable during 12 months of follow-up (79.02 ± 64.39 vs. 44.71 ± 58.01; *p* = 0.004). However, plasma SN concentrations (97.62 ± 75.18 vs. 77.06 ± 33.22; *p* = 0.192) were higher in patients who achieved CE (all-cause mortality + all- cause readmission) than those who did not, but without statistical significance. To determine cut-offs of those parameters, we used ROC analysis which confirmed the statistically significant role of NT-proBNP, age and SN, as three the strongest factors predicting death of CHF patients during 12 months of follow-up (Table 5).

**Table 5.** Parameters of diagnostic usefulness obtained in ROC analysis for biomarkers discriminating HFrEF patients who died during one-year follow-up and those who survived observation period.

Biomarker	Cut-offs	AUC, 95%CI	P
Age (years)	63	0.769; 0.659-0.879	< 0.001
BMI (kg/m^2^)	18.5	0.290; 0.154-0.427	0.003
Secretoneurin (pmol/l)	34.5	0.760; 0.669-0.852	<0.001
NT-proBNP (pg/ml)	2375	0.806; 0.712-0.901	<0.001
Creatinine (mg/dl)	1.11	0.715; 0.587-0.843	< 0.001
Hematocrit (%)	45.2	0.301; 0.157-0.445	0.007
Platelets count (1000/mm3)	319	0.337-0.178-0.496	0.044
Neutrophiles (%)	68.7	0.722; 0.600-0.844	< 0.001

AUC: area under curve; CI: confidence interval; ROC: receiver operating characteristic curve; BMI: body mass index; NT-proBNP: N-terminal fragment of B-type natriuretic propeptide; p: probability; AUC: area under the curve.

When comparing data on SN and NT-proBNP, we noticed different dynamics of these markers in the groups of patients with diabetes (Table 6). Additionally, we noted lower SN concentrations among men, but it should be emphasized that the share of females in our research group was much smaller (Table 7).

**Table 6.** T-test for secretoneurin and NT-proBNP grouping patients with diabetes mellitus.

variable	Test t; diabetes mellitus								
	DM+	DM-	t	df	p	n1	n0	Sd1	Sd0
secreto­neurin (pmol/l)	75.662	41.799	3.043	120	0.003	50	72	80.589	41.150
NT-proBNP (pg/ml)	5608.520	5970.875	−0.253	120	0.800	50	72	5955.269	8801.171

NT-proBNP: N-terminal fragment of B-type natriuretic propeptide; DM: diabetes mellitus; n1: group with stable heart failure with reduced ejection fraction; n0: group with exacerbated heart failure with reduced ejection fraction, Sd: standard deviation; p: probability.

**Table 7.** T-test for secretoneurin grouping patients according to gender.

variable	Test-t; gender								
	male	female	t	df	p	male	female	Sd1	Sd2
secretoneurin (pmol/l)	49.555	77.222	−2.057	120	0.042	95	27	60.322	66.264

df: degrees of freedom; Sd: standard deviation; p: probability.

### 4.3. Survival analysis

**Figure 2.**
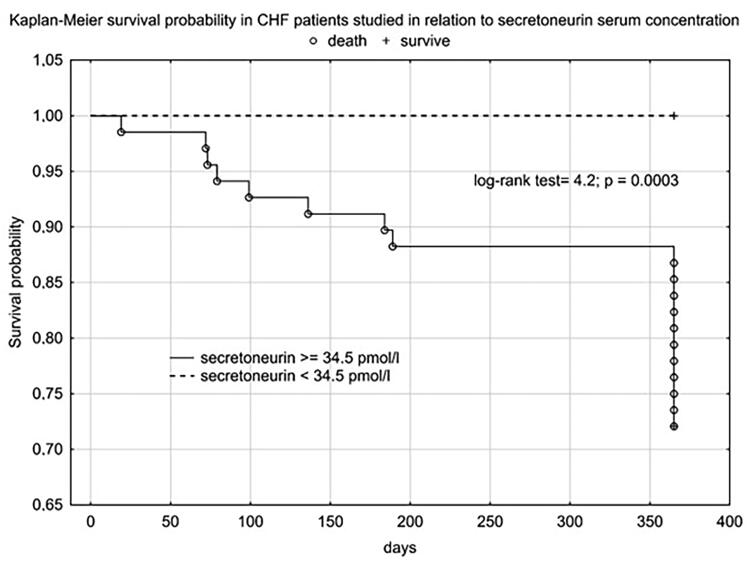
Kaplan Meier’s plot for HFrEF patients survival in relation to plasma SN concentration.

Using cut-offs values obtained in ROC analysis we performed Kaplan Meier analysis, which confirmed the clinical importance of use of, i.a., SN as a prognostic factor for one-year mortality among CHF patients (Figure 2).

**Figure 3.**
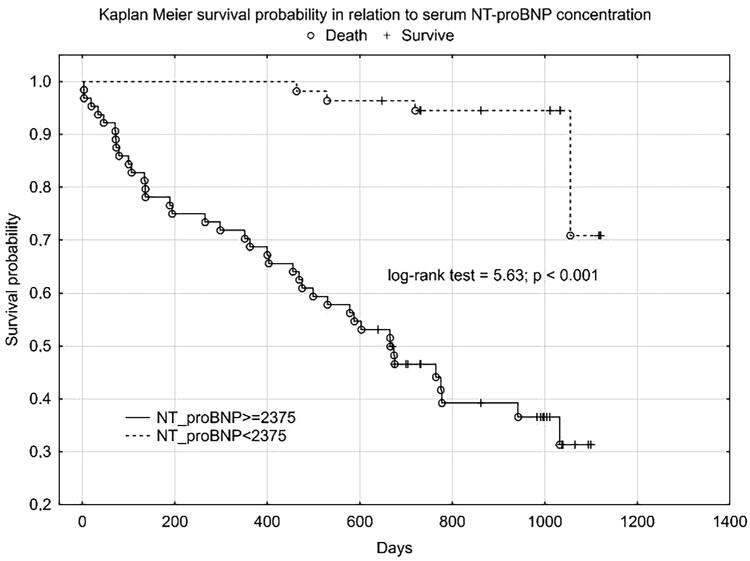
Kaplan Meier’s plot for HFrEF patients survival in relation to plasma NT-proBNP concentration.

**Figure 4.**
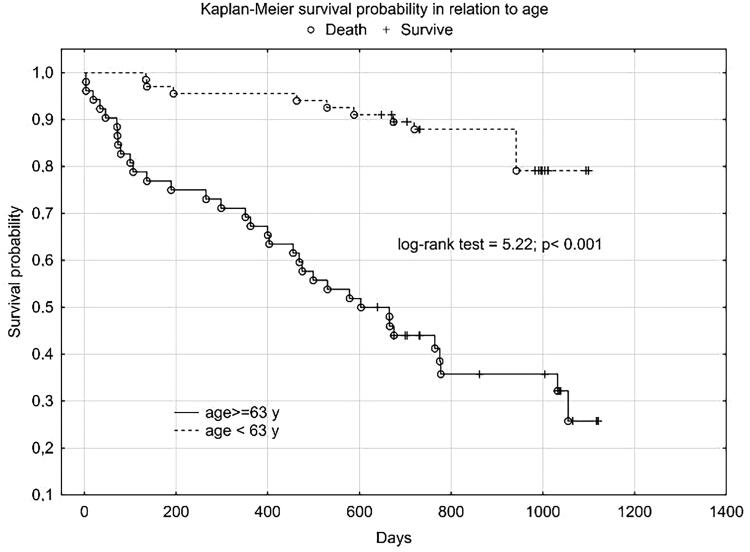
Kaplan Meier’s plot for HFrEF patients survival in relation to age.

We obtained a similar graph for NT-proBNP (Figure 3) and age **(**Figure 4).

## 5. Discussion

Since the incorporation of natriuretic peptide assessment into routine clinical practice, many reliable markers of heart failure have been tested with varying results [9–17]. A multicenter study from 2021 assessed SN for analytical quality by evaluating within subject variation (CVI), between subject variation (CVG), reference change values (RCV) and index of individuality (II) [18]. SN is characterized by low values of the above parameters, which indicates its high potential as a prognostic biomarker and monitoring the course of the disease [18]. Considering the high residual risk, reflecting the multifaceted pathophysiology of heart failure, even with the great clinical role of natriuretic peptides, they seem to be insufficient. Simultaneous measurement of multiple markers can provide a broader insight into various aspects pathophysiology of HFrEF and increase the prognostic value of performed tests, which should be the basis for modern risk stratification. Such an approach will help refine therapeutic strategies and allow treatment to be tailored to individual clinical and biochemical profile of each patient with CHF. Changes in SN concentrations are the result of a number of interrelated pathomechanisms involved in CHF, such as cardiomyocyte Ca2+ dysregulation, chronic inflammation, excessive activation of the immune and sympathetic systems and the resulting consequences.

All biological actions of SN are not yet fully understood or discovered. It is speculated that SN acts through membrane receptors [1]. It has been reported that it is a G protein-coupled receptor [19]. In addition to regulating calcium metabolism by influencing CaMKII and ryanodine receptors, SN may act directly at various subcellular levels. It induces nitric oxide synthase 3 (NOS3) [20], and inhibits tight junctions between endothelial cells, which increases their permeability to nitric oxide (NO) [21, 22]. Endothelium-dependent cardioprotection also includes cyclooxygenase (COX) activation and an increase in prostacyclin production [20]. By activating phosphoinositide-3 kinase/protein kinase B and mitogen-associated protein kinase (MAPK), SN exerts anti-apoptotic effects on endothelial cells [23]. This peptide promotes transendothelial leukocyte migration and adhesion in a manner comparable to tumor necrosis factor alpha [24]. In a mouse model of myocardial infarction (MI) using SN in gene therapy, a reduction in scar formation and unfavorable cardiac remodeling was achieved, as well as an increase in coronary angiogenesis in the borderline zone of MI, and improvement in left ventricular function. Researchers showed that SN acted as an angiogenic cytokine - it increased the binding of vascular endothelial growth factor (VEGF) to endothelial cells and the binding of VEGF to its co-receptor neuropilin 1 (NRP-1). In endothelial cells, SN stimulated fibroblast growth factor receptor-3 (FGFR3) and insulin-like growth factor-1 (IGF-1) receptor and in coronary smooth muscle cells SN stimulated the VEGF-1 receptor and FGFR3 [25]. In another study SN attenuated isoproterenol-induced myocardial cell hypertrophy by inhibiting ROS production and induced superoxide dismutase and catalase activity [26]. It reduced natriuretic peptide levels and interstitial fibrosis as well as heart weight to body weight ratio [26]. The pleiotropic profile of action SN is an exponent of the fact that after action factor leading to a change in the strength of myocardial contraction, there must be many compensatory mechanisms that are interrelated and interdependent, and the circulatory system decompensates after reaching a certain critical point. Higher plasma concentrations of this peptide may indirectly reflect the arrhythmogenic potential, calcium-dependent cardiac remodelling and, as our results suggest, the level of hemodynamic instability. Currently, there is a lack of data that shows the superiority of the use of SN over currently used markers in specific clinical situations. Our preliminary data indicate that SN, like NT-proBNT, increases with NYHA class, but shows differences among patients with diabetes mellitus. Due to the limited research group, larger studies should be carried out to confirm the obtained results. Confirmation of these results in further studies could promote the use of SN over NT-proBNP in specific clinical situations.

A review of the existing literature indicates that this manuscript is the first in the world to examine the prognostic value of SN in a group of HFrEF patients and as one of the first in patients with HF. We were the first to compare SN concentrations between stable and exacerbated HF patients, including individuals with de novo HF. This is the only study on SN with a homogeneous research group in the context of LVEF, while other researchers did not specify LVEF, or included patients with HFrEF, HFmrEF (heart failure with mildly reduced ejection fraction), even HFpEF (heart failure with preserved ejection fraction) [3, 6] at the same time.

The analysis of the collected data indicates that SN concentration depends on the severity of HFrEF. The risk of all-cause mortality and rehospitalization among the study patients was associated with some biomarkers, of which the plasma SN concentration seems promising and worth further evaluation. ROC analysis among all analyzed markers confirmed the statistically significant role of NT-proBNP, age and SN as the three strongest predictors of death in patients with CHF during 12 months of observation.

Plasma SN concentration increased with NYHA class, we obtained a statistically significant correlation between SN and NT-proBNP concentration and the correlation coefficient for NYHA class was higher for SN than for NT-proBNP. In the 12-month follow-up, high SN levels were noted for all three endpoints. Despite not reaching statistical significance for the CE (rehospitalization + death from any cause), we obtained a clear trend of higher SN values in the group that achieved CE. Statistical significance and high SN values were obtained independently for the CE components. As expected, we observed a significant difference in SN levels between these groups - clinically stable and exacerbated. Patients with an exacerbation on the day of inclusion in the study were significantly older, had lower TAPSE, ang higher levels of NT-proBNP, SN and troponin T. The discrepancy in marker levels in relation to the clinical course suggests that SN is a dynamic cardiovascular marker that reflects clinical condition and the severity of hemodynamic instability rather than a chronic heart failure process. According to Kaplan-Meier and the data in Tables 4 and 5, the prognostic value for selected endpoints in the 1-year follow-up is similar for SN and NT-proBNP. This should be compared with the availability and economic considerations of the determinations made between these markers. SN appears to be a new protective mediator activated in the most severely ill patients with HF and a biomarker that reflects CV pathophysiology not covered by established risk markers.

Higher SN values have also been reported in critical conditions such as severe sepsis and septic shock [7], acute heart failure, out-of-hospital cardiac arrest (OHCA) due to arrhythmia [6] and in patients with catecholaminergic polymorphic ventricular tachycardia (CPVT) [5]. The concentration of SN normalized after cardioversion, which may support the hypothesis that the release of SN from damaged cardiomyocytes in situations of deep systemic stress and myocardial pathology [6]. Similar observations regarding SN levels and previously used CV markers were observed in patients after cardiac arrest (*n* = 155) caused by ventricular arrhythmias [5]. Contrary to the SN levels on admission, which dropped rapidly after resuscitation, on the 1st day of hospitalization, NT-proBNP levels increased [admission- mean: 62 (range: 21-152) pmol/L vs 1st day of hospitalization- mean: 128 (range: 61-261) pmol/L] and hs-TnT did not differ significantly. The researchers noted higher SN levels at admission, 24 and 48 h of hospitalization in the group that survived the follow-up period relative to those who died. SN on admission- mean: 154, range: 120-185 (pmol/L) vs mean 163, range: 136-240 (pmol/L); *p* = 0.03; 24h- mean: 102, range: 76-148 (pmol/L) vs mean 114, range: 80-150 *p* = 0,17; 48h: mean: 102, range: 73-136 (pmol/L) vs mean: 121, range: 83-160 pmol/L *p* = 0.08. Researchers believe that this is consistent with the model of SN as an endogenous CaMKII inhibitor, where overstimulation leads to arrhythmogenic calcium release, as the risk of new ventricular arrhythmia events is highest in the early post-hospital period. In another study, 143 patients were hospitalized for acute heart failure. During follow up, SN levels were significantly correlated with mortality (*n* = 66 death, median 776 days; hazard ratio [lnSN]: 4.63; 95% confidence interval: 1.93 to 11.11; *p* = 0.001) [6].

In HF patients scheduled for cardiac surgery, preoperative plasma SN concentrations may be a complementary risk stratification marker to identify patients at higher risk of death. Among HF patients scheduled for surgery due to aortic stenosis, those who died- mean: 156, range: 133–209 (pmol/l); survivors- mean: 140, range: 116–155 (pmol/l) *p* = 0.007 [3]. Similarly, in patients undergoing coronary artery bypass grafting (CABG), plasma SN levels were decreased post-procedure in survivors and increased in non-survivors at follow-up [mean:173, range: 129-217 (pmol/L) vs mean:143, range: 111-173 (pmol/L); *p* < 0.001] [3]. A limitation in the use of SN as a prognostic marker may be the so far poorly understood influence of other systems on the concentration of SN in CV diseases. However, current data support the release of SN into the circulation from the neuroendocrine system, including from nerve fibers running along the coronary vessels, and from injured cardiomyocytes, based on increased SN immunoreactivity in the myocardium, but not in other organs [27]. Future studies should focus on measuring SN among similar cohorts of HF patients, taking into account their clinical parameters, variability of SN metabolism, medical history and pharmacotherapy received, including new generation drugs [28–31]. This action will allow the standardization of SN levels as a useful prognostic biomarker in CHF that reflects additional cardiovascular pathophysiology not covered by established risk markers. The limitation of our study is the relatively small study population and the limited possibility of comparing the obtained results to other clinical trials due to the lack of studies analyzing the prognostic significance of SN in chronic heart failure, including HFrEF [32, 33].

## 6. Conclusion

Plasma SN concentration showed relationships with severity of HFrEF. The risk all-cause mortality and all cause readmission in HFrEF patients studied was related to some biomarkers, of which plasma SN concentration seems to be promising and worth of further evaluation factor, despite of the power of its diagnostic yield was lower than NT-proBNP and patients age. Patients with high SN levels may benefit from systematic follow-up and may be candidates for more aggressive treatment.

## Data Availability

All data used to support the finding of this study are available from the corresponding author upon request.
